# The Activity of Red Nigerian Propolis and Some of Its Components against *Trypanosoma brucei* and *Trypanosoma congolense*

**DOI:** 10.3390/molecules28020622

**Published:** 2023-01-07

**Authors:** Samya S. Alenezi, Naif D. Alenezi, Godwin U. Ebiloma, Manal J. Natto, Marzuq A. Ungogo, John O. Igoli, Valerie A. Ferro, Alexander I. Gray, James Fearnley, Harry P. de Koning, David G. Watson

**Affiliations:** 1 Strathclyde Institute of Pharmacy and Biomedical Science, University of Strathclyde, 161 Cathedral Street, Glasgow G4 0RE, UK; 2 Institute of Infection, Immunity and Inflammation, College of Medical, Veterinary and Life Sciences, University of Glasgow, Glasgow G12 8TA, UK; 3 School of Health and Life Sciences, Teesside University, Middlesbrough TS1 3BX, UK; 4 Department of Veterinary Pharmacology and Toxicology, Faculty of Veterinary Medicine, Ahmadu Bello University, Zaria 810107, Kaduna State, Nigeria; 5 Department of Chemical Sciences, Pen Resource University, Gombe-Bauchi Road, Gombe 771104, Lafiyawo, Nigeria; 6 BeeVital, Whitby YO22 5JR, North Yorkshire, UK

**Keywords:** red Nigerian propolis, isoflavonoids, *Trypanosoma brucei*, *Trypanosoma congolense*

## Abstract

Propolis is a resin that is gathered by bees from exudates produced by various plants. Its exact chemical composition depends on the plants available near the hive. Bees use propolis to coat the surfaces of the hive, where it acts as an anti-infective. Regardless of the chemical composition of propolis, it is always anti-protozoal, probably because protozoan parasites, particularly *Lotmarium passim*, are widespread in bee populations. The protozoa *Trypanosoma brucei* and *T. congolense* cause disease in humans and/or animals. The existing drugs for treating these diseases are old and resistance is an increasingly severe problem. The many types of propolis present a rich source of anti-trypanosomal compounds—from a material gathered by bees in an environmentally friendly way. In the current work, red Nigerian propolis from Rivers State, Nigeria was tested against *T. brucei* and *T. congolense* and found to be highly active (EC_50_ 1.66 and 4.00 µg/mL, respectively). Four isoflavonoids, vestitol, neovestitol, 7-methylvestitol and medicarpin, were isolated from the propolis. The isolated compounds were also tested against *T. brucei* and *T. congolense*, and vestitol displayed the highest activity at 3.86 and 4.36 µg/mL, respectively. Activities against drug-resistant forms of *T. brucei* and *T. congolense* were similar to those against wild type.

## 1. Introduction

Bees gather propolis from the exudates of buds or bark of a variety of plants; the variety in the source leads to a wide chemical diversity in its constituents. Propolis is used by bees to coat surfaces within the hive and as an anti-infective agent [1,2] against microbial infections. Activity has been found against a variety of bee pathogens including *Varroa* mites [3], *Paenibacillus larvae* [4] and *Nosema ceranae* [5]. Although bees are not known to ingest propolis as such, many of the flavonoids present in temperate propolis are found in honey and may originate from propolis gathered by the bees [6]. The presence of a propolis coating in the beehive has been found to stabilize and improve the honeybee microbiome [7]. There have been no direct studies on the efficacy of propolis in reducing the burden of protozoa in beehives. However, it is well established that the trypanosomatid *Lotmarium passim* is widespread in beehives [8,9,10]. Propolis is frequently anti-protozoal and particularly active against kinetoplastids [11]. The extent to which protozoal infection is a threat to bee health has not been clearly delineated, although the major bee pathogen *N. ceranae* was once classified as a protozoan and there is evidence that protozoal infection may contribute to winter colony collapse [12].

Since propolis contains a multitude of chemical components in a complex mixture, its biological activity and pharmacological properties might be expected to be broad [1,2]. Propolis and some compounds purified from it have been shown to have promising activity against a number of protozoal species, particularly the kinetoplastid species *Trypanosoma* and *Leishmania* [11,13,14,15,16,17,18,19]. Diseases caused by parasitic kinetoplastids remain a problem worldwide. Examples are human African trypanosomiasis (HAT) and animal African trypanosomiasis (AAT), which occur in Africa; surra and dourine, livestock and equine diseases, respectively, that are prevalent in the Middle East and Southern Asia; and Chagas disease in South and Central America. These are all caused by *Trypanosoma* species [20,21]. In addition, the closely related *Leishmania* parasites cause a variety of diseases throughout the world, particularly cutaneous and visceral leishmaniasis [21,22]. Chemotherapy is still important for the control of most parasitic diseases, including trypanosomiasis and leishmaniasis, as no vaccines are available and vector control is often rudimentary or impracticable in the affected regions. The treatment of HAT or AAT is currently based on a few drugs that were developed decades ago. However, the current frontline drugs are quite toxic and, in most cases, require parenteral administration. Furthermore, resistance to current drugs by trypanosomes is another threat to effective chemotherapy [22,23,24].

Propolis samples from Nigeria have previously been investigated for their major constituents. One study identified phenolic compounds including calycosin, liquiritigenen, pinocembrin, vestitol, medicarpin, prenylnaringenin, and macarangin, in addition to xanthones and some triterpenes in the samples [16]. The results of in vitro assays with Nigerian propolis indicated high activity against *T. b. brucei* and other protozoa [13,16]. A recent study also investigated the chemical composition of Nigerian propolis by means of combined chromatographic and spectroscopic techniques and identified isoflavonoids, diarylpropanes and flavanones. The antioxidant activity and α-amylase and α-glucosidase inhibition of Nigerian propolis have also been tested and the results suggested that Nigerian propolis could be a good raw material for nutraceuticals and food products [25]. Other studies found a potent inhibitory activity by Nigerian propolis against *Helicobacter pylori*, highlighting its potential as an anti-bacterial agent [26]. In the current study, we have further examined red Nigerian propolis, which we previously found had promising activity against *T. b. brucei* [13], and the compounds isolated from it against *T. b. brucei* and against *T. congolense* including multi-drug-resistant strains of both species.

## 2. Results

### 2.1. Characterization of the Ethanol Extract of Nigerian Propolis by ^1^H Nuclear Magnetic Resonance Spectroscopy (NMR)

The proton NMR (Supplementary Materials Figure S1) of the ethanolic extract of the Nigerian propolis showed the presence of triterpenoids with methyl group signals observed between δ_H_ 0.18 and 2.56 ppm. The triterpenoids had to be of the cycloartane type as there were cyclopropane protons between 0.30 and 0.49 ppm, which could imply mangiferolic or isomangiferolic acid or cycloartenol. There was no evidence for the presence of flavonoids containing a C-5-OH chelated to a C-4-C=O. The NMR spectrum also showed the presence of oxygenated methine or methylene protons between 4.00 and 5.20 ppm (although there was an overlap with some shielded double-bond protons in this region) indicating the presence of lignans or flavans and isoflavans. The presence of aromatic and olefinic protons between 6.12 and 7.14 ppm was confirmation of aromatic moieties in flavonoids, lignans, flavans and/or isoflavans. There was also evidence for the presence of phenolic hydroxyls (between 3.00 and 9.69 ppm) and methoxy protons at 3.66 and 4.00 ppm implying the alkylation of hydroxyl groups in the aromatic rings.

### 2.2. Characterization of Red Nigerian Propolis Subfractions Using Liquid Chromatography–Mass Spectrometry (LC-MS) and ^1^HNMR

**Scheme 1:**
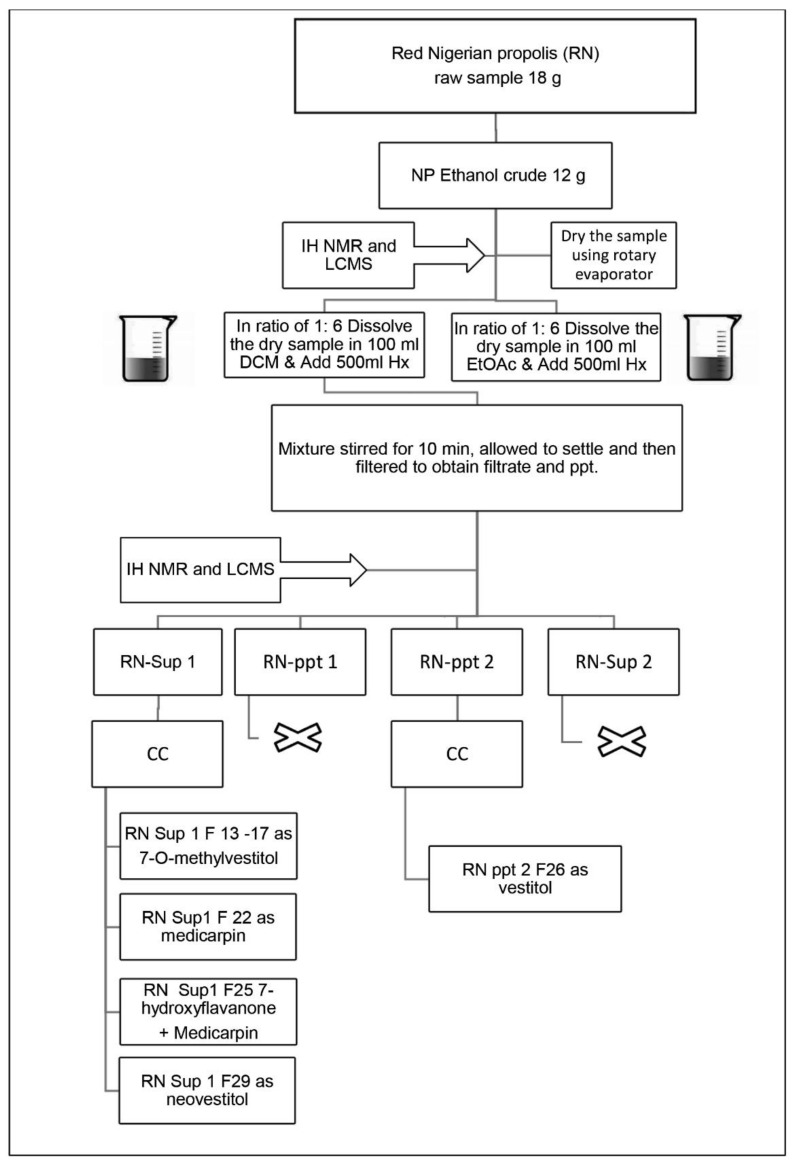
Procedure used for the fractionation of extracts from red Nigerian propolis. (sup = supernatant; ppt = precipitate.) DCM = dichloromethane. A total of 80 fractions (F1-F 80) of 20 mL each were collected.

The ethanolic extract of the red Nigerian propolis was further fractionated as described in Scheme 1. The ethanolic crude extract of the Nigerian sample was subfractionated into four fractions called RN sup1, RN ppt1, RN ppt2 and RN sup2. Preliminary screening of the fractions was performed via the facilitated selection of fractions rich in phenolics for further fractionation. The ^1^HNMR spectra of these are shown in Figures S2–S5; the RN Sup1 and RN ppt2 were selected for further fractionation since they appeared to be rich in phenolic compounds. The LC-MS profiling results for the RN sup1 and RN ppt are shown in Tables S1 and S2 and suggested a high content of flavonoids, lignans and/or other phenolic compounds, with varying degrees of oxygenation.

### 2.3. Isolation of Compounds from RN sup1 and RN ppt 2

#### 2.3.1. Isolation of 7-O-Methylvestitol

7-O-methylvestitol (Figure S6) was obtained from RN sup1 following column chromatography (CC). Fractions 13–17 were combined based on thin-layer chromatography (TLC) analysis. The LC-MS chromatogram showed a peak at 18.8 min with a [M-H]^−^ ion at *m*/*z* 285.1126 corresponding to C_17_H_17_O_4_ (Calc 285.1127) in the negative mode. The NMR data are shown in Table S3 and the structure of 7-O-methylvestitol was confirmed by comparison with the literature [27] data shown in the table.

#### 2.3.2. Isolation of Neovestitol

Neovestitol (Figure S7) was isolated from RN sup 1. LC-MS (ESI) showed a peak at 13.4 min with a [M-H]^−^ ion at *m*/*z* 271.0970 (C_16_H_15_O_4_, calc 271.0970). This confirmed a molecular formula of C_16_H_16_O_4_.

The NMR data are given in Table S4 and the structure of neovestitol was confirmed by comparison with the literature [28] data shown in the table.

#### 2.3.3. Characterization of 7-Hydroxyflavanone in a Mixture with Medicarpin

A fraction containing 7-hydroxy flavanone (Figure S8) was isolated from RN Sup1 by CC. LC-MS data were composed of two main peaks: one at 14.1 min and an ion [M+H]^+^ at *m*/*z* 241.0848 (C_15_H_13_O_3_) (Calc 241.0864) and another peak at 15.5 min and an [M+H]^+^ ion at *m*/*z* 271.0956 corresponding to C_16_H_15_O_4_ (Calc. 271.0970) due to medicarpin.

The NMR data are given in Table S5 and the structure of 7-hydoxy flavanone was confirmed by comparison with the literature [29] data shown in the table.

#### 2.3.4. Isolation of Medicarpin

Medicarpin (Figure S9) was isolated from RN ppt 2 by CC. The LC-MS chromatogram showed a peak at 15.4 min with a [M+H]^+^ ion at *m*/*z* 271.0955 (C_16_H_15_O_4_) (Calc 271.0970), corresponding to the molecular formula C_16_H_14_O_4_.

The NMR data are given in Table S6 and the structure of medicarpin was confirmed by comparison with the literature [27,30] values shown in the table.

#### 2.3.5. Isolation of Vestitol

Vestitol (Figure S10) was isolated from RN ppt2 by CC. In LC-MS ESI, a peak at 13.6 min was observed with a [M-H]^−^ ion observed at *m*/*z* 271.0971 corresponding to C_16_H_15_O_4_ (Calc 271.0970), which confirmed the molecular formula as C_16_H_16_O_4_. The NMR data are shown in Table S7 and the structure of vestitol was confirmed by comparison with the literature [27] values shown in the table.

### 2.4. In Vitro Anti-Trypanosomal Activity and Cross-Resistance Studies of Red Nigerian Propolis Extracts and Its Fractions

The crude extract from red Nigerian propolis, its fractions, and isolated compounds were tested against BSF *T. b. brucei* s427 WT and *T. b. brucei* B48, and against BSF *T. congolense* IL3000 WT and *T. congolense* 6C3, in order to assess their antiparasitic activity and the potential for cross-resistance with key existing drugs. All EC_50_ values are shown in Table 1 and Table 2 as averages, in μg/mL and μM, of at least 3 independent determinations. RN Sup1 showed relatively high activity against *T. b. brucei* with an EC_50_ value of 1.66 ± 0.13 μg/mL, while the purified compounds exhibited lower activities than the RN-Sup1. A notable observation was that *p* values for all the fractions, apart from for RN sup2, were (*p* > 0.05; Student’s unpaired, two-tailed *t*-test), and all displayed a resistance factor (RF) of <2 compared to a 225-fold resistance for pentamidine (*p* < 0.0001). We conclude that these compounds are not cross-resistant with pentamidine, and that also means no cross-resistance with the melaminophenyl arsenical class of trypanocides [31,32]. This conforms to the current models of drug resistance in trypanosomes as the resistance to both drugs is coupled to loss of the TbAT1 aminopurine transporter and the aquaporin AQP2, and the compounds here lack the requisite recognition motifs for these carriers [33].

EC_50_ values for *T. congolense* IL3000 WT and its derived diminazene-resistant cell line 6C3 are shown in Table 2. The most active fractions were RN-Sup1 and vestitol with EC_50_ 4.00 and 4.36 μg/mL, respectively. Neither the RN extracts nor purified compounds displayed cross-resistance with diminazene, which is the first-line treatment against *T. congolense* infection in domestic animals [34], as no significant differences with strain 6C3 were observed (*p* > 0.05; Student’s unpaired, two-tailed *t*-test) with resistance factors (RF) within 2-fold compared to 14.4-fold resistance for diminazene (*p* < 0.0002). The assay’s precision was achieved for all samples with <30% RSD.

### 2.5. In Vitro Cytotoxicity of Nigerian Propolis Extracts and Its Fractions to Mammalian Cells

The red Nigerian propolis crude extract, its fractions, and purified compounds were tested in vitro for their cytotoxicity activities against human cell line U937 [35]. The cell viability was determined using a resazurin-based assay. The toxicity results in Table 3 showed that RN pp2 F22 displayed the lowest EC_50_ value. The fractions RN sup1, RN ppt2, and RN sup2 showed low toxicity to these cells. Thus, RN fractions displayed a good level of selectivity against kinetoplastid parasites compared to the mammalian cells. The lowest SI value was obtained was 5.4 and the highest was 51.8.

## 3. Discussion

We have previously isolated several compounds from red Nigerian propolis and tested them against *T. b. brucei* [13,16]. Here, we extend the study to additional compounds and to screening against *T. congolense* as well. Of the compounds isolated in the current paper, only vestitol was isolated in the previous work and was tested against *T. b. brucei*. Thus, medicarpin, neovestitol and 7-methylvestitol had not yet been tested against either *Trypanosoma* species. From the previous work, it was apparent that the highest activities against *T. b. brucei* were obtained for the prenylated flavonoids [13,16]. In our earlier study, we tested multiple Nigerian propolis samples and found that red Nigerian propolis samples from Rivers State displayed the highest activity against *T. b. brucei* [16].

Some of the fractions’ extracts, prepared according to Scheme 1, displayed higher activity against *T. b. brucei* than any of the compounds isolated from the mixtures. This suggests that either there are unknown compounds present in the mixtures that have higher activity than any of those that were actually isolated, or there has to be significant synergy between some of the active components in the mixtures. Distinguishing between these two possibilities would require much additional research.

The isoflavonoids isolated from red Nigerian propolis exhibited a similar or slightly higher activity against *T. b. brucei* than the flavonoids that were previously isolated from temperate propolis samples [17]. Vestitol and neovestitol are isomers and vestitol has slightly higher activity. However, this cannot be linked to the position of the free hydroxyl groups since the 7-methoxy analogue has a similar activity to neovestitol although it is methoxylated at both the C-7 and C-4 positions. All the fractions and isolated compounds had a slightly lower activity against the pentamidine-resistant *T. b. brucei* line B48 in the test employed, which used an incubation time with the drug of 48 h. As the B48 strain lacks the TbAT1/P2 and TbAQP2 transporters [36], it is very possible that this changes its response to various stress factors just sufficiently to explain this minimal shift in EC_50_; certainly, the small differences cannot be categorized as cross-resistance and we have seen similar small differences with unrelated compound series [37]. The compounds and fractions were also tested against *T. congolense*. Again, the crude extract was more active than the isolated compounds. Of the isolated compounds, vestitol had the highest activity. There were no marked differences between the activity of the compounds against the wild type and resistant lines of *T. congolense*. The isolated compounds all exhibited low levels of toxicity against mammalian cells, having selectivity indices of 9 or above when comparing toxicity against U937 cells with toxicity against *T. b. brucei*. The isoflavanoid compounds found in Nigerian red propolis have all been found in Brazilian red propolis and, recently, their plant source has been identified [38]. It is apparent that red Nigerian propolis has much in common with red Brazilian propolis. Thus, is distinctively different from other African propolis samples which tend to contain high levels of triterpenes and diterpenes [14,15,16].

The consistent observation of anti-protozoal activity of propolis does suggest that evolutionary pressure has impelled bees to collect plant exudates that yield a propolis that is effective in controlling protozoal infections. Although the protozoa *L. passim* has only been characterized in recent years, it is now well-established that it is prevalent in honeybee populations [39]. The protozoa colonize the gut of the bee and are transmitted to other members of the hive via feces and there is a possibility that it can be transmitted beyond the hive via contact with flowers visited by infected bees [40]. It has not been firmly established that *L. passim* has a negative impact on bee health but there is a growing body of evidence that this is the case [40,41]. Coating of the surface of the beehive with propolis could serve to reduce the level of transmission of *L. passim* and/or other infective organisms on or in the hive. Bees have not been observed to ingest propolis as such, but certainly in the case of temperate propolis, many of the flavonoids found in propolis are also found in honey, and it has been suggested that over 50% of the flavonoid content of many European honeys is derived from propolis [42]. This strongly indicates that propolis is mixed into the honey and this might be a strategy for reducing levels of protozoa in the gastrointestinal tract since the flavonoids in temperate propolis also have anti-protozoal properties [19]. Indeed, in areas lacking poplar trees, which are the source of temperate propolis, the typical temperate propolis flavonoids are only rarely found in honey from such areas [42]. African honeys have not been profiled in detail for their flavonoid content, so it is not known whether the isoflavonoids found in red Nigerian propolis are found in the corresponding honeys.

Given its promising anti-trypanosomal activity, red Nigerian propolis could provide potential lead compounds for treating human protozoal diseases. The flavonoids in temperate propolis are extensively metabolized [43,44]. In the case of the isoflavonoids themselves, it is not clear whether or not the activity would be retained after metabolism. The major flavonoids in temperate propolis are known to be completely converted to sulfates and glucuronides in vivo. The metabolites may not retain their anti-protozoal effects. In previous research, it was observed that RN propolis significantly increased the survival of rats infected with *T. b. brucei*, and reduced the pathology of the infection [45].

*T. congolense* and *T. b. brucei* are among the trypanosome species responsible for causing AAT in sub-Saharan Africa, whereas trypanosome species including *T. evansi*, *T. vivax* and *T. equiperdum* have spread AAT increasingly to South America and Southeast Asia [20], causing great economic damage and impacting food security. No new drugs for treating AAT have been developed for over 60 years and resistance to the existing drugs is a serious problem [24,46]. The availability of locally produced remedies based on natural products would be attractive. Since the unfractionated red Nigerian propolis extract has high anti-trypanosomal activity, it might be possible to administer crude extract or fractions as a treatment. Additionally, since the propolis extracts and active compounds are active against both diminazene-resistant *T. congolense* and pentamidine/arsenical-resistant *T. b. brucei*, it might be possible to administer a treatment based on red Nigerian propolis where trypanocide resistance is a problem. Propolis is almost always anti-protozoal and, thus, through the many types available, could provide a rich source of anti-protozoal drugs. In comparison to the extraction of phytochemicals from plants, propolis provides a concentrated source of phytochemicals which the bee has gathered in an environmentally friendly way.

## 4. Materials and Methods

### 4.1. General

The propolis sample was collected by Prof. John Igoli from a beekeeper in the Island of Bonny (4°26′ N 7°10′ E) in Rivers State, Nigeria in 2016. It had a reddish-brown color, was friable, and was without a strong odor.

Solvents, reagents, and other consumables were obtained from Sigma Aldrich, Fisher Scientific, BioWhittaker, or Merck.

### 4.2. Preliminary Treatment of the Propolis Sample

The propolis sample was treated as shown diagrammatically in Scheme 1.

Approximately 30 g of the propolis sample was extracted thrice under sonication (Clifton ultrasonic bath, Fisher Scientific, Loughborough, UK), with 150 mL of ethanol at room temperature for 60 min. The extracts were combined, and the solvent was evaporated using a rotary evaporator (Buchi, VWR, Leicestershire, UK), and the residue was weighed and yielded about 12 g of extract. The extract was divided into two equal portions by re-dissolving in ethanol and evaporating to dryness. One portion was redissolved in dichloromethane and then 600 mL hexane was added and the mixture was stirred for 10 min and allowed to settle, and the precipitate was filtered off (treatment 1). The other portion was dissolved in ethyl acetate and 600 mL of hexane was added and the mixture was stirred for 10 min and allowed to settle, and the precipitate was filtered off (treatment 2). The four resulting fractions were dried on a rotary evaporator and weighed. The filtrate from treatment 1 (RN sup1) and the precipitate from treatment 2 (RN ppt2) were selected for further fractionation.

### 4.3. Column Chromatography

Then, separately, about 1 g of RN sup1 and 1 g of RN ppt2 were dissolved in 5 mL of ethyl acetate and mixed with 6 g of silica gel in a beaker and allowed to dry in a fume hood. A glass column was packed with 60 g of silica gel 60 (0.063–0.2 mm, Sigma Aldrich) in hexane. The dry adsorbed extract was added directly onto the column and eluted using 200 mL each of hexane, ethyl acetate and methanol mixtures as follows: hexane:ethyl acetate (80:20), hexane/ethyl acetate (60:40), hexane/ethyl acetate (40:60), hexane:ethyl acetate (20:80), ethyl acetate and then ethyl acetate/methanol (80:20), ethyl acetate/methanol (60:40), ethyl acetate/methanol (40:60), ethyl acetate/methanol (20:80) and finally methanol. We collected 20 mL fractions, yielding 80 fractions from the column. All the isolated compounds eluted in the first 30 fractions. The compounds eluted as follows: from RN sup1 7-O methyl vestitol (hexane:ethyl acetate 60:40 in fractions 13–17); medicarpin (hexane:ethyl acetate 40:60 fraction 22); hydroxyflavanone/medicarpin mixture (hexane:ethyl acetate 40:60 fraction 25); neovestitol (hexane:ethyl acetate 40:60 fraction 29); and from RN ppt2 vestitol (hexane:ethyl acetate 40:60 fraction 26). The purity of the isolated compounds was confirmed by reversed-phase HPLC with evaporative light-scattering detection (ELSD).

### 4.4. LC-MS Analysis

All samples and fractions were dissolved in methanol to give a concentration of 1 mg/mL and were analyzed using an Agilent 1100 HPLC linked to a Shodex ELSD. An ACE C-18 column (150 × 3 mm, 3 μm) with a mobile phase of water (A) and acetonitrile (B) and a flow rate of 0.3 mL/min was used with the following gradient: 25% B for 30 min, 5 min 100% B and 5 min 25% B, injecting 10 μL of sample solution. High-resolution mass spectra were obtained by running the samples on a Dionex 3000 HPLC connected to an Orbitrap Exactive mass spectrometer (ThermoFisher, Hemel Hempstead, UK). The MS detection range was from 100 to 1200 *m*/*z* and the scanning was performed under electrospray ionization polarity-switching mode. The needle voltages were set at −4.0 kV (negative) and 4.5 kV (positive) and sheath and auxiliary gases were at 50 and 17 arbitrary units, respectively. Separation was performed on an ACE C18 column (150 × 3 mm, 3 μm) with 0.1% *v*/*v* formic acid in water as mobile phase A and 0.1% *v*/*v* formic acid in acetonitrile as B at a flow rate of 0.300 mL/min using the gradient described for HPLC–ELSD.

### 4.5. NMR

About 5–10 mg of the fractions obtained from CC purification was dissolved in CDCl_3_, DMSO-*d_6_*, or acetone-*d_6_* and spectra were acquired using a Bruker AVIII-HD-500 NMR spectrometer.

### 4.6. Determination of the Cytotoxic Effect of PNG Extract and Its Purified Compounds on U937 Mammalian Cells

U937 cells (European Collection of Cell Cultures Cat. No. 85011440, supplied by Sigma Aldrich, Dorset, UK) were cultured as described previously [35]. U937 cells were grown to log phase at 37 °C and harvested at a density of 1 × 10^5^ cells/mL in a 96-well plate (TPP, Trasadingen, Switzerland). Aliquots of 100 µL/well of the cells were added and the plate was incubated for 24 h at 37 °C, 5% CO_2_, and 100% humidity. A 2-fold serial dilution of the test compound was carried out in growth medium in another 96-well plate, and 100 μL of each dilution was then transferred to the cultured cells using a multichannel pipette, followed by another incubation for 24 h. Resazurin dye (Sigma Aldrich, Dorset, UK) was then added at a final concentration of 10% (*v*/*v*) and the plates were incubated for a further 24 h, after which fluorescence was measured using a Wallac Victor 2 microplate reader (λ Ex/Em: 560/590 nm). The compounds and fractions were tested in triplicate, and cell viability was expressed as a percentage of the drug-free control. The resulting data were analyzed using GraphPad Prism 8 to obtain dose–response curves and corresponding mean inhibitory concentration (EC_50_) values.

### 4.7. Anti-Protozoal Assay

The extract and the purified compounds were cultured and tested against *T. b. brucei*, and *T*. *congolense* exactly as described previously, using a resazurin-based assay [16,17]. The *T. b. brucei* strains were a standard drug-sensitive lab strain, Lister 427 (wild-type) [47] and the derived cell line B48 was developed from the wild-type by gene deletion of the drug transporter TbAT1 followed by in vitro adaptation to pentamidine [48], leading to the further loss of the gene encoding TbAQP2 [49], rendering it highly resistant to the diamidine and melaminophenyl arsenical classes of trypanocides. The *T. congolense* strains were the lab strain IL3000 and its diminazene-adapted clone 6C3 [50]. All EC_50_ values presented are the average of at least three independent determinations.

## Figures and Tables

**Table 1:** EC_50_ values of red Nigerian propolis, its fractions, and isolated compounds on *T. b. brucei s427* wild-type (WT) and multi-drug-resistant strain B48 (AVG ± SEM; *n* = 3).

Samples	*T. b. brucei* WT		*T. b. brucei* B48		Comparison	
	EC_50_ (μg/mL)	EC_50_ (μM)	EC_50_ (μg/mL)	EC_50_ (μM)	RF	*p* Value
RN sup 1	1.66 ± 0.13	-	2.22 ± 0.36		1.34	0.062
7-O-methyl vestitol	3.44 ± 0.66	12.0 ± 2.3	4.66 ± 1.03	16.3 ± 3.6	1.36	0.159
medicarpin	7.93 ± 0.56	29.3 ± 2.1	8.90 ± 0.57	33.0 ± 2.1	1.12	0.102
7-hydroxyflavanone/ Medicarpin	7.73 ± 1.52	-	9.14 ± 0.72	-	1.18	0.219
neovestitol	5.69 ± 0.94	18.6 ± 3.1	7.88 ± 1.54	29.0 ± 5.7	1.39	0.103
RN ppt 2	2.53 ± 0.47	-	2.93 ± 0.71	-	1.16	0.454
vestitol	3.86 ± 0.54	14.2 ± 2.0	4.75 ± 0.43	17.4 ± 1.6	1.23	0.088
RN sup2	2.51 ± 0.58	-	4.92 ± 0.46	-	1.96	0.005
pentamidine	-	0.0022 ± 0.0019	-	0.49 ± 0.07	225	0.0002

RF, resistance factor, the ratio of EC_50_(B48)/EC_50_(WT). The two EC_50_ values were compared for statistical difference using an unpaired, two-tailed *t*-test.

**Table 2:** EC_50_ values of red Nigerian propolis its fractions and isolated compounds on *T. congolense* IL300, and the *T. congolense* cell line 6C3, resistant to diminazene (AVG ± SEM, *n* = 3).

Samples	*T. congolense* IL300		*T. congolense* 6C3		Comparison	
	EC_50_ (μg/mL)	EC_50_ (μM)	EC_50_ (μg/mL)	EC_50_ (μM)	RF	*p* Value
RN sup 1	4.00 ± 0.72	-	4.68 ± 0.50	-	1.17	0.250
7-O-methyl vestitol	12.1 ± 1.6	42.3 ± 5.5	12.5 ± 1.8	43.7 ± 6.5	1.03	0.790
medicarpin	8.70 ± 0.91	32.2 ± 3.4	7.45 ± 1.04	27.6 ± 3.8	0.86	0.195
7-hydroxyflavanone/ medicarpin	7.78 ± 0.85	-	7.93 ± 1.08	-	1.02	0.854
neovestitol	7.51 ± 1.6	27.6 ± 5.8	7.10 ± 0.49	26.1 ± 1.8	0.95	0.621
RN ppt 2	6.70 ± 1.8	-	6.74 ± 0.95	-	1.01	0.968
vestitol	4.36 ± 0.46	16.0 ± 1.7	4.47 ± 0.75	16.4 ± 2.8	1.02	0.844
RN sup2	14.7 ± 2.6	-	13.2 ± 3.3	-	0.90	0.571
diminazene	-	0.26 ± 0.03	-	3.69 ± 0.83	14.4	0.002

**Table 3:** EC_50_ of cytotoxicity of red Nigerian propolis, its fractions, and isolated compounds against U937 cells (AVG ± SEM; *n* = 3).

Samples	U937		
	EC_50_ (μg/mL)	EC_50_ (μM)	SI
RN sup 1	86.0 ± 7.3	-	51.8
7-*O*-methyl vestitol	44.4 ± 3.4	155 ± 12	12.9
medicarpin	42.4 ± 6.4	157 ± 24	5.34
7-hydroxyflavanone/ medicarpin	46.8 ± 6.8	-	6.06
neovestitol	51.2 ± 5.7	188 ± 6	9.00
RN ppt 2	130 ± 5.1	-	51.2
vestitol	65.0 ± 4.1	238 ± 15	16.8
RN sup2	120 ± 8.3	-	47.6

SI, the ratio of the EC_50_ values of U937 and *T. b. brucei* WT cells.

## Data Availability

All relevant data are contained in the manuscript and Supplemental Information.

## Supplementary Materials

The following supporting information can be downloaded at: https://www.mdpi.com/article/10.3390/molecules28020622/s1, Figure S1: ^1^H NMR (500 MHz) spectrum of an ethanolic extract of RN propolis in DMSO-d_6_; Figure S2: ^1^H NMR (500 MHz) of RN-Sup 1 in DMSO-d_6_; Figure S3: ^1^H NMR (500 MHz) of RN-ppt1 in DMSO-*d_6_*; Figure S4: ^1^H NMR (500 MHz) of RN-Sup 2 in DMSO-*d_6_*; Figure S5: ^1^H NMR (500 MHz) of RN-ppt2 in DMSO-*d_6_*; Table S1: High-resolution MS profiling of RN-Sup1 crude using negative-ion masses; Table S2: High-resolution MS profiling of RN-ppt 2 using negative-ion masses; Figure S6: Structure of 7-O-methylvestitol; Table S3: ^1^H (400 MHz), ^13^C (100 MHz) chemical shifts in CDCl_3_ 7-O-methylvestitol; Figure S7: Structure of 2′,4′-dihydroxy-7-methoxyisoflavan (neovestitol); Table S4: ^1^H (400 MHz), ^13^C (100 MHz) data for neovestitol in Acetone-d_6_; Figure S8: Structure of 7-hydroxyflavanone; Table S5: ^1^H (400 MHz), ^13^C (100 MHz) chemical shifts in CDCl_3_ for 7-Hydroxyflavanone; ^13^C NMR (100 MHz) spectra of 7-hydroxyflavanone/medicarpin mixture in CDCl_3_. The signals due to 7-hydroxy flavanone have been numbered; Figure S9: Structure of medicarpin; Table S6: ^1^H (400 MHz), ^13^C (100 MHz) chemical shifts in CDCl_3_ for medicarpin_3_; Figure S10: Structure of vestitol; Table S7: ^1^H (400 MHz), ^13^C (100 MHz) chemical shifts in CDCl_3_ for vestitol.

## Sample Availability

Samples of the compounds can be obtained from the authors subject to availability.

## References

[1] Bankova V., Popova M., Trusheva B. (2018). The phytochemistry of the honeybee. Phytochemistry.

[2] Siheri W., Alenezi S., Tusiimire J., Watson D.G. (2017). The chemical and biological properties of propolis. Bee Products-Chemical and Biological Properties.

[3] Pusceddu M., Annoscia D., Floris I., Frizzera D., Zanni V., Angioni A., Satta A., Nazzi F. (2021). Honeybees use propolis as a natural pesticide against their major ectoparasite. Proc. R. Soc. B.

[4] Wilson M., Brinkman D., Spivak M., Gardner G., Cohen J. (2015). Regional variation in composition and antimicrobial activity of US propolis against *Paenibacillus larvae* and *Ascosphaera apis*. J. Invertebr. Pathol..

[5] Mura A., Pusceddu M., Theodorou P., Angioni A., Floris I., Paxton R.J., Satta A. (2020). Propolis consumption reduces Nosema ceranae infection of European honey bees (*Apis mellifera*). Insects.

[6] Habryka C., Socha R., Juszczak L. (2020). The effect of enriching honey with propolis on the antioxidant activity, sensory characteristics, and quality parameters. Molecules.

[7] Saelao P., Borba R.S., Ricigliano V., Spivak M., Simone-Finstrom M. (2020). Honeybee microbiome is stabilized in the presence of propolis. Biol. Lett..

[8] Regan T., Barnett M.W., Laetsch D.R., Bush S.J., Wragg D., Budge G.E., Highet F., Dainat B., de Miranda J.R., Watson M. (2018). Characterisation of the British honey bee metagenome. Nat. Commun..

[9] Ravoet J., Schwarz R.S., Descamps T., Yañez O., Tozkar C.O., Martin-Hernandez R., Bartolomé C., De Smet L., Higes M., Wenseleers T. (2015). Differential diagnosis of the honey bee trypanosomatids *Crithidia mellificae* and *Lotmaria passim*. J. Invertebr. Pathol..

[10] Quintana S., Plischuk S., Brasesco C., Revainera P., García M.L.G., Bravi M.E., Reynaldi F., Eguaras M., Maggi M. (2021). *Lotmaria passim* (Kinetoplastea: Trypanosomatidae) in honey bees from Argentina. Parasitol. Int..

[11] Ebiloma G.U., Ichoron N., Siheri W., Watson D.G., Igoli J.O., De Koning H.P. (2020). The strong anti-kinetoplastid properties of bee propolis: Composition and identification of the active agents and their biochemical targets. Molecules.

[12] Ravoet J., Maharramov J., Meeus I., De Smet L., Wenseleers T., Smagghe G., De Graaf D.C. (2013). Comprehensive bee pathogen screening in Belgium reveals *Crithidia mellificae* as a new contributory factor to winter mortality. PLoS ONE.

[13] Omar R.M., Igoli J., Gray A.I., Ebiloma G.U., Clements C., Fearnley J., Edrada Ebel R.A., Zhang T., De Koning H.P., Watson D.G. (2016). Chemical characterisation of Nigerian red propolis and its biological activity against *Trypanosoma brucei*. Phytochem. Anal..

[14] Siheri W., Zhang T., Ebiloma G.U., Biddau M., Woods N., Hussain M.Y., Clements C.J., Fearnley J., Ebel R.E., Paget T. (2016). Chemical and antimicrobial profiling of propolis from different regions within Libya. PLoS ONE.

[15] Siheri W., Ebiloma G.U., Igoli J.O., Gray A.I., Biddau M., Akrachalanont P., Alenezi S., Alwashih M.A., Edrada-Ebel R., Muller S. (2019). Isolation of a novel flavanonol and an alkylresorcinol with highly potent anti-trypanosomal activity from Libyan propolis. Molecules.

[16] Omar R., Igoli J.O., Zhang T., Gray A.I., Ebiloma G.U., Clements C.J., Fearnley J., Ebel R.E., Paget T., De Koning H.P. (2017). The chemical characterization of Nigerian propolis samples and their activity against *Trypanosoma brucei*. Sci. Rep..

[17] Alotaibi A., Ebiloma G.U., Williams R., Alenezi S., Donachie A.-M., Guillaume S., Igoli J.O., Fearnley J., De Koning H.P., Watson D.G. (2019). European propolis is highly active against trypanosomatids including *Crithidia fasciculata*. Sci. Rep..

[18] Cavalcante G.M., Camara C.A., Silva E.M.S.D., Santos M.S., Leite A.B., Queiroz A.C., Evelyn Da Silva A., Araújo M.V., Alexandre-Moreira M.S., Silva T.M.S. (2021). Leishmanicidal activity of propolis collected in the Semiarid Region of Brazil. Front. Pharmacol..

[19] Alotaibi A., Ebiloma G.U., Williams R., Alfayez I.A., Natto M.J., Alenezi S., Siheri W., AlQarni M., Igoli J.O., Fearnley J. (2021). Activity of compounds from temperate propolis against *Trypanosoma brucei* and *Leishmania mexicana*. Molecules.

[20] Giordani F., Morrison L.J., Rowan T.G., De Koning H.P., Barrett M.P. (2016). The animal trypanosomiases and their chemotherapy: A review. Parasitology.

[21] Simarro P.P., Diarra A., Ruiz Postigo J.A., Franco J.R., Jannin J.G. (2011). The human African trypanosomiasis control and surveillance programme of the World Health Organization 2000–2009: The way forward. PLoS Negl. Trop. Dis..

[22] Field M.C., Horn D., Fairlamb A.H., Ferguson M.A., Gray D.W., Read K.D., De Rycker M., Torrie L.S., Wyatt P.G., Wyllie S. (2017). Anti-trypanosomatid drug discovery: An ongoing challenge and a continuing need. Nat. Rev. Microbiol..

[23] Stuart K., Brun R., Croft S., Fairlamb A., Gürtler R.E., McKerrow J., Reed S., Tarleton R. (2008). Kinetoplastids: Related protozoan pathogens, different diseases. J. Clin. Investig..

[24] De Koning H.P. (2020). The drugs of sleeping sickness: Their mechanisms of action and resistance, and a brief history. Trop. Med. Infect. Dis..

[25] Alaribe C.S., Esposito T., Sansone F., Sunday A., Pagano I., Piccinelli A.L., Celano R., Cuesta Rubio O., Coker H.A., Nabavi S.M. (2021). Nigerian propolis: Chemical composition, antioxidant activity and α-amylase and α-glucosidase inhibition. Nat. Prod. Res..

[26] Chinwe S.A., Rastrelli L., Sunday A., Emmanuel O., Sola I., Herbert C. (2013). Anti-*Helicobacter pylori* activities of Nigerian propolis (Bee Product). Planta Med..

[27] Piccinelli A.L., Campo Fernandez M., Cuesta-Rubio O., Márquez Hernández I., De Simone F., Rastrelli L. (2005). Isoflavonoids isolated from Cuban propolis. J. Agric. Food Chem..

[28] Franchin M., Colón D.F., da Cunha M.G., Castanheira F.V., Saraiva A.L., Bueno-Silva B., Alencar S.M., Cunha T.M., Rosalen P. (2016). Neovestitol, an isoflavonoid isolated from Brazilian red propolis, reduces acute and chronic inflammation: Involvement of nitric oxide and IL-6. Sci. Rep..

[29] Kostrzewa-Susłow E., Janeczko T. (2012). Microbial transformations of 7-hydroxyflavanone. The Sci. World J..

[30] Yang X., Zhao Y., Hsieh M.T., Xin G., Wu R.T., Hsu P.L., Horng L.Y., Sung H.C., Cheng C.H., Lee K.H. (2017). Total synthesis of (+)-medicarpin. J. Nat. Prod..

[31] Munday J.C., Settimo L., De Koning H.P. (2015). Transport proteins determine drug sensitivity and resistance in a protozoan parasite, *Trypanosoma brucei*. Front. Pharmacol..

[32] Carter N.S., Barrett M.P., De Koning H.P. (1999). A drug resistance determinant from *Trypanosoma brucei*. Trends Microbiol..

[33] Bray P.G., Barrett M.P., Ward S.A., De Koning H.P. (2003). Pentamidine uptake and resistance in pathogenic protozoa. Trends Parasitol..

[34] Anene B.M., Ezeokonkwo R.C., Mmesirionye T.I., Tettey J.N.A., Barrett M.P., De Koning H.P. (2006). A diminazene resistant strain of *Trypanosoma brucei brucei* isolated from a dog is cross-resistant to pentamidine in experimentally infected albino rats. Parasitology.

[35] Passmore J.S., Lukey P.T., Ress S.R. (2001). The human macrophage cell line U937 as an in vitro model for selective evaluation of mycobacterial antigen-specific cytotoxic T-cell function. Immunology.

[36] Alghamdi A.H., Munday J.C., Campagnaro G.D., Gurvic D., Svensson F., Okpara C.E., Kumar A., Quintana J., Abril M.E.M., Milić P. (2020). Positively selected modifications in the pore of TbAQP2 allow pentamidine to enter *Trypanosoma brucei*. eLife.

[37] Ebiloma G.U., Ayuga T.D., Balogun E.O., Gil L.A., Donachie A., Kaiser M., Herraiz T., Inaoka D.K., Shiba T., Harada S. (2018). Inhibition of trypanosome alternative oxidase without its N-terminal mitochondrial targeting signal (ΔMTS-TAO) by cationic and non-cationic 4-hydroxybenzoate and 4-alkoxybenzaldehyde derivatives active against *T. brucei* and *T. congolense*. Eur. J. Med. Chem..

[38] Ccana-Ccapatinta G.V., Mejía J.A., Tanimoto M.H., Groppo M., Carvalho J.C., Bastos J.K. (2020). *Dalbergia ecastaphyllum* (L.) Taub. and *Symphonia globulifera* Lf: The botanical sources of isoflavonoids and benzophenones in Brazilian red propolis. Molecules.

[39] Arismendi N., Castro M.P., Vargas M., Zapata C., Riveros G. (2022). The trypanosome *Lotmaria passim* prevails in honey bees of different ages and stages of development. J. Apicult. Res..

[40] Gómez-Moracho T., Buendía-Abad M., Benito M., García-Palencia P., Barrios L., Bartolomé C., Maside X., Meana A., Jiménez-Antón M.D., Olías-Molero A.I. (2020). Experimental evidence of harmful effects of Crithidia mellificae and Lotmaria passim on honey bees. Int. J. Parasitol..

[41] Liu Q., Lei J., Darby A.C., Kadowaki T. (2020). Trypanosomatid parasite dynamically changes the transcriptome during infection and modifies honey bee physiology. Commun. Biol..

[42] Tomás-Barberán F.A., Ferreres F., García-Vignera C., Tomás-Lorente F. (1993). Flavonoids in honey of different geographical origin. Z. Lebensm. Unters. Forsch..

[43] Rofaidi M.A., Alotaibi A., Alqarni A., Alghamdi A., Fearnley J., Watson D.G. (2020). A preliminary study of the absorption and metabolism of temperate propolis by human subjects. J. Food Nutr. Metab..

[44] Bloor S.J., Mitchell K.A. (2021). Metabolic products of European-type propolis. Synthesis and analysis of glucuronides and sulfates. J. Ethnopharmacol..

[45] Nweze N.E., Okoro H.O., Al Robaian M., Omar R.M., Tor-Anyiin T.A., Watson D.G., Igoli J.O. (2017). Effects of Nigerian red propolis in rats infected with *Trypanosoma brucei brucei*. Comp. Clin. Pathol..

[46] Delespaux V., De Koning H.P., Jäger T., Koch O., Flohe L. (2013). Transporters in anti-parasitic drug development and resistance. Trypanosomatid Diseases: Molecular Routes to Drug Discovery.

[47] De Koning H.P., MacLeod A., Barrett M.P., Cover B., Jarvis S.M. (2000). Further evidence for a link between melarsoprol resistance and P2 transporter function in African trypanosomes. Mol. Biochem. Parasitol..

[48] Bridges D., Gould M.K., Nerima B., Mäser P., Burchmore R.J.S., De Koning H.P. (2007). Loss of the High Affinity Pentamidine Transporter is responsible for high levels of cross-resistance between arsenical and diamidine drugs in African trypanosomes. Mol. Pharmacol..

[49] Munday J.C., Eze A.A., Baker N., Glover L., Clucas C., Aguinaga Andrés D., Natto M.J., Teka I.A., McDonald J., Lee R.S. (2014). *Trypanosoma brucei* aquaglyceroporin 2 is a high-affinity transporter for pentamidine and melaminophenyl arsenic drugs and the main genetic determinant of resistance to these drugs. J. Antimicrob. Chemother..

[50] Carruthers L.V., Munday J.C., Ebiloma G., Steketee P., Jayaraman S., Campagnaro G.D., Ungogo M.A., Lemgruber L., Donachie A.-M., Rowan T.G. (2021). Diminazene resistance in *Trypanosoma congolense* is not caused by reduced transport capacity but associated with reduced mitochondrial membrane potential. Mol. Microbiol..

