# Supplementary material for: The Activity of Red Nigerian Propolis and Some of Its Components against Trypanosoma brucei and Trypanosoma congolense
Source: Molecules. 2023 Jan 7;28(2):622. doi: 10.3390/molecules28020622 (PMC9860874; doi:10.3390/molecules28020622)
Supplement: Supplementary file 1 [file molecules-28-00622-s001.zip › molecules-2079637-supplementary.pdf]

The activity of red Nigerian propolis and some of its components against *Trypanosoma brucei* and *Trypanosoma congolense*

Samya S. Alenezi[1], Naif D. Alenezi[1], Godwin U. Ebiloma[2,4] Manal J. Natto[2], Marzuq A. Ungogo[2,4], John O. Igoli[2,3], Valerie A. Ferro[1], Alexander I. Gray[1], James Fearnley[4], Harry P. de Koning*[2], David G. Watson[1]*

Supplementary material

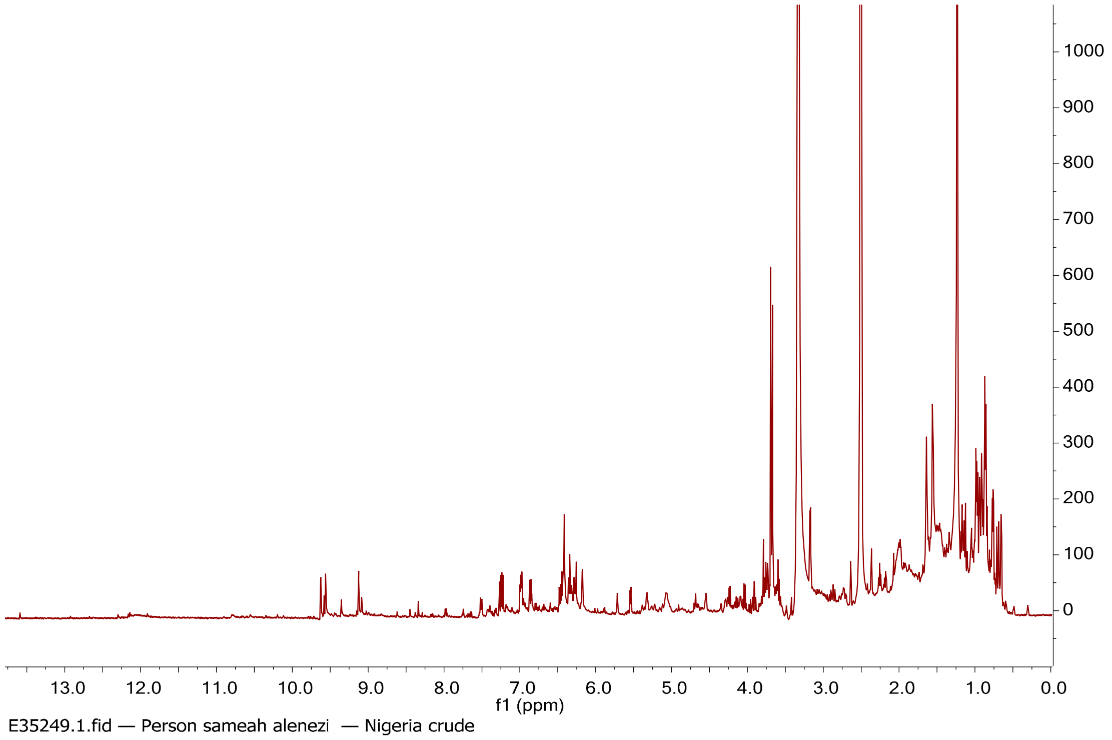

Figure S1: ^{1}H NMR (500 MHz) spectrum of the ethanol extract of RN propolis in DMSO-d_6

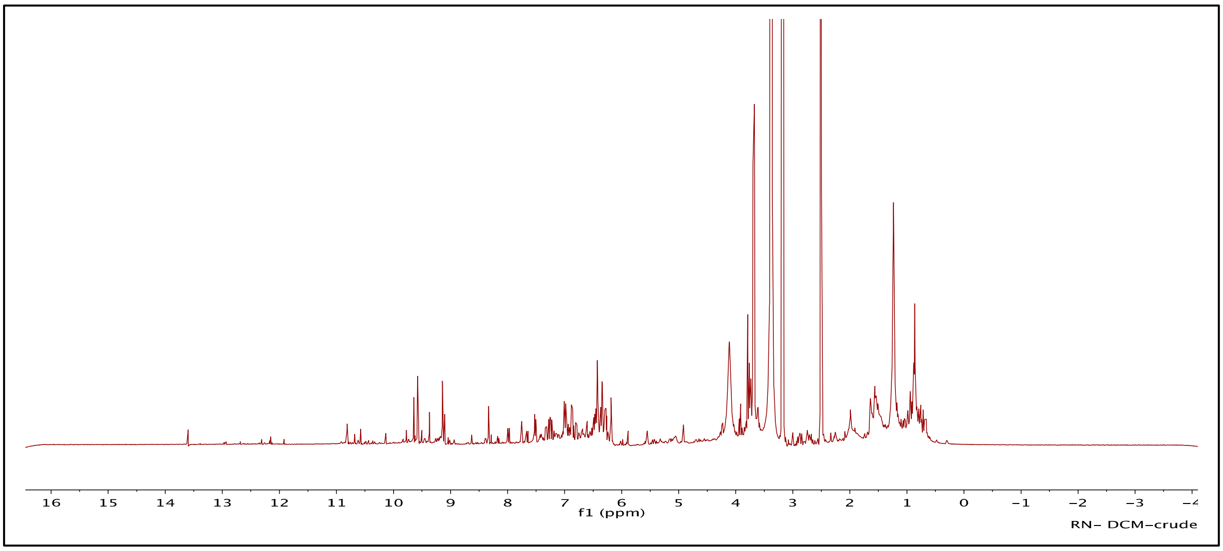

Figure S2: ^{1}H NMR (500 MHz) spectrum of RN-Sup 1 in DMSO-d_6

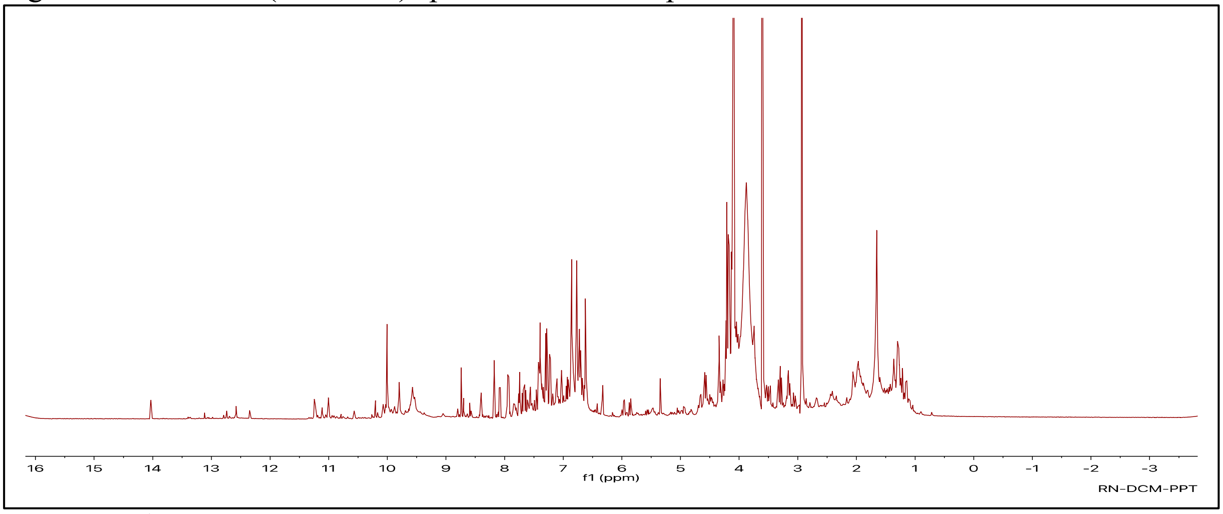

Figure S3: ^{1}H NMR (500 MHz) spectrum of RN-ppt1 in DMSO-d_6

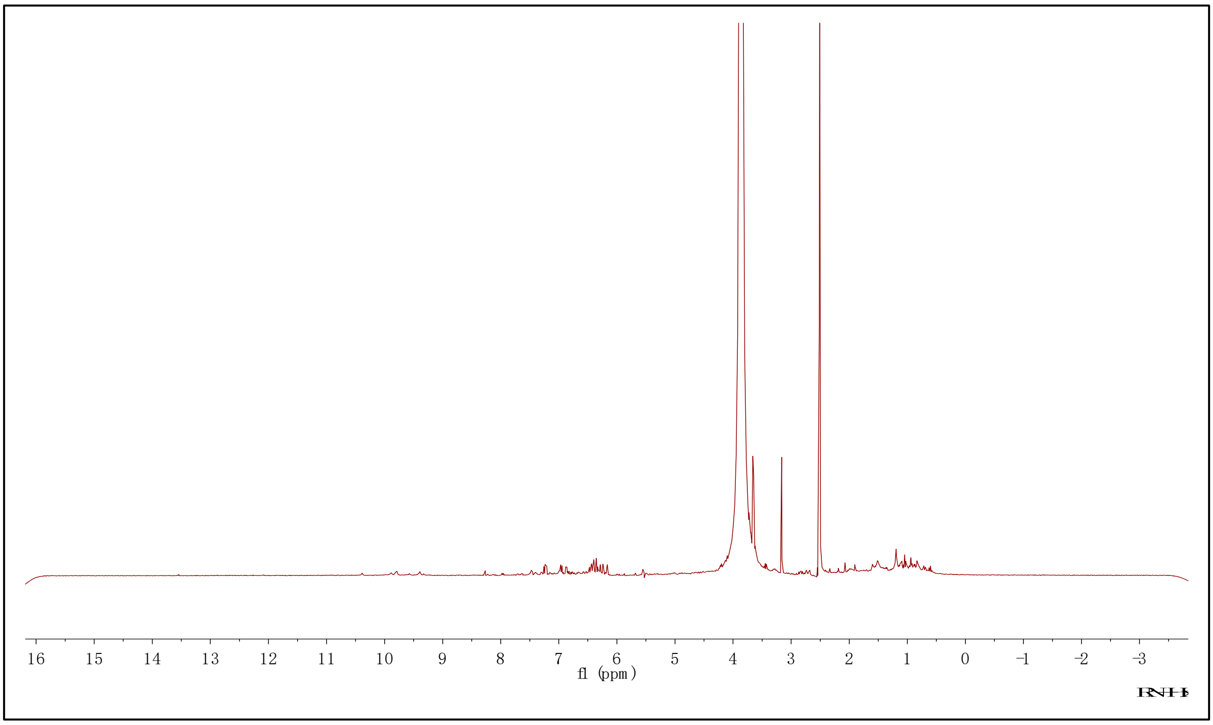

Figure S4 ¹H NMR (500 MHz) spectrum of RN-Sup 2 in DMSO-d_6

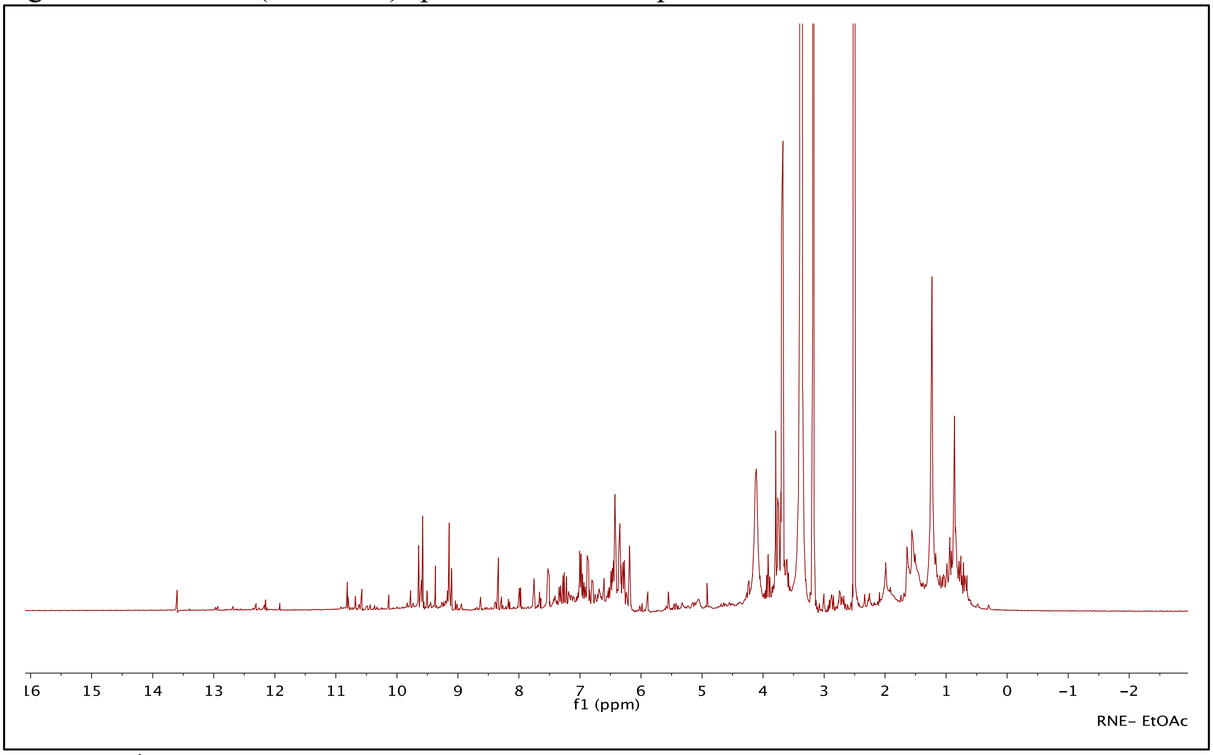

Figure S5 ¹H NMR (500 MHz) spectrum of RN-ppt2 in DMSO-d_6

Table S1 High resolution MS profiling of RN-Sup1 crude using negative ion masses

Peak no	*RT (min)*	*M-1*	*Formula*	*RDB*	*Delta (ppm)*	*Intensity*

1	9.01	315.0867	$C_{17}H_{15}O_6$	10.5	-2.25	1.78x 10^6
2	11.24	271.0608	$C_{16}H_{11}O_5$	10.5	-1.61	1.67x 10^6
3	12.83	255.0659	$C_{15}H_{11}O_4$	10.5	-1.65	2.21x10^6
		301.0712	$C_{16}H_{13}O_6$	10.5	-1.96	
4	13.41	267.0657	$C_{16}H_{11}O_4$	11.5	-2.29	3.91x10^6
5	13.66	267.0660	$C_{16}H_{11}O_4$	11.5	-1.31	3.51x10^6
6	14.53	271.0971	$C_{16}H_{15}O_4$	9.5	-1.85	2.34x10^6
7	15.87	271.0971	$C_{16}H_{15}O_4$	9.5	-1.85	1.82x10^6
8	16.18	269.0814	$C_{16}H_{13}O_4$	10.5	-2.13	1.11x10^7
9	17.72	255.0656	$C_{15}H_{11}O_4$	10.5	-2.12	6.68x10^6
10	17.96	266.0579	$C_{16}H_{10}O_4$	12.0	-2.02	8.20x10^6
11	18.82	285.1128	$C_{17}H_{17}O_4$	9.5	-1.446	5.24x10^5
12	20.31	240.0422	$C_{14}H_8O_4$	11.0	-2.49	7.35x10^5
			$C_{16}H_{15}O_4$	9.5	-0.97	

Table S2: High resolution MS profiling of RN-ppt 2 using negative ion masses

Peak no	*RT (min)*	*M-1*	*Formula*	*RDB*	*Delta (ppm)*	*Intensity*
1	6.72	287.0562	$C_{15}H_{11}O_6$	10.5	0.45	7.93x10^6
		329.1607	$C_{16}H_{25}O_7$	4.5	0.41	
2	15.29	323.1291	$C_{20}H_{19}O_4$	11.5	0.55	2.37x10^7
3	15.84	439.1763	$C_{25}H_{27}O_7$	12.5	0.24	1.36x10^7
4	16.53	455.1715	$C_{25}H_{27}O_8$	12.5	0.88	1.88x10^7
5	17.52	339.1240	$C_{20}H_{19}O_5$	12.5	0.48	2.76x10^7
6	17.91	383.1141	$C_{21}H_{19}O_7$	12.5	1.16	9.84x10^7
7	18.56	395.1138	$C_{22}H_{19}O_7$	13.5	0.36	2.32x10^7
		339.1240	$C_{20}H_{19}O_5$	11.5	0.66	
8	19.29	381.1346	$C_{25}H_{21}O_6$	12.5	0.57	5.77x10^7
		319.2280	$C_{20}H_{31}O_3$	5.5	0.41	
9	19.82	417.2288	$C_{24}H_{33}O_6$	8.5	1.33	1.72x10^7
		485.3277	$C_{30}H_{45}O_5$	8.5	0.87	
10	20.78	339.1240	$C_{20}H_{19}O_5$	11.5	0.57	2.36x10^7
		483.2025	$C_{27}H_{31}O_8$	12.5	0.54	
11	21.44	367.1190	$C_{21}H_{19}O_6$	12.5	0.76	7.76x10^7
		339.1241	$C_{20}H_{19}O_5$	11.5	1.04	
12	23.20	423.1818	$C_{25}H_{27}O_6$	12.5	1.20	3.62x10^7
13	23.52	423.1815	$C_{25}H_{27}O_6$	12.5	0.49	1.46x10^7
		323.1291	$C_{20}H_{19}O_4$	11.5	0.64	
14	24.92	407.1865	$C_{25}H_{27}O_5$	12.5	0.30	6.65x10^7

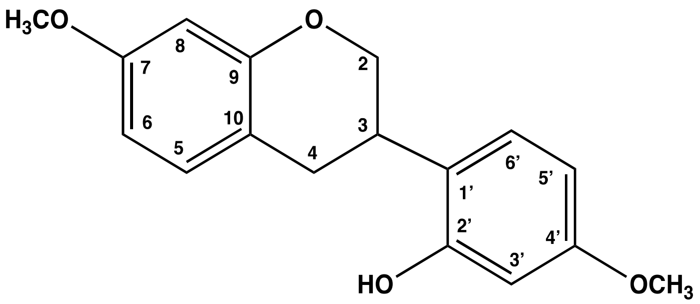

Chemical Formula: $C_{17}H_{18}O_4$

Figure S6: Structure of 7-*O*-methylvestitol

Table S3 ^{1}H (400MHz), ^{13}C (100MHz) chemical shifts inCDCl3 7-O-methylvestitol. *CD3OD

Position	^{1}H δ ppm (mult, *J* Hz)	^{13}C δ ppm (mult)	^{1}H δ ppm* From literature [1]	^{13}C δ ppm* From literature[1]
2	4.34 (1H, ddd, 10.5, 3.5, 2.0) 4.05 (1H, m)	69.9 (CH_2)	4.22, 3.85	55.6
3	3.51 (1H, m)	31.7 (CH)	3.41	33.1
4	3.01 (1H, ddd, 15.7, 10.5, 1.1) 2.91 (1H, ddd, 15.8, 5.4, 1.9)	30.3 (CH_2)	2.7, 2.86	31.3
5	7.01 (1H, d, 8.5)	128.2 (CH)	6.9	131.2
6	6.47 (1H, dd, 8.4, 2.6)	107.2 (CH)	6.47	107.9
7	-	159.0 (C)	-	160.8
8	6.36 (1H, d, 2.5)	102.1 (CH)	6.38	102.5
9	-	155.0 (C)	-	157.3
10	-	119.9 (C)	-	116.0
1′	-	114.4 (C)	-	121.3
2′	-	154.2 (C)	-	156.2
3′	6.42 (1H, d, 2.6)	101.4 (CH)	6.4	102.5
4′	-	159.3 (C)	-	160.5
5′	6.47 (1H, dd, 8.4, 2.6)	106.1 (CH)	6.27	105.8
6′	6.98 (1H, dd, 8.3, 1.0)	130.2 (CH)	6.85	128.8
7-OCH_3	3.77 (3H, s)	55.3 (CH_3)	3.41	55.6
4′- OCH_3	3.77 (3H, s)	55.3 (CH_3)	3.41	55.6

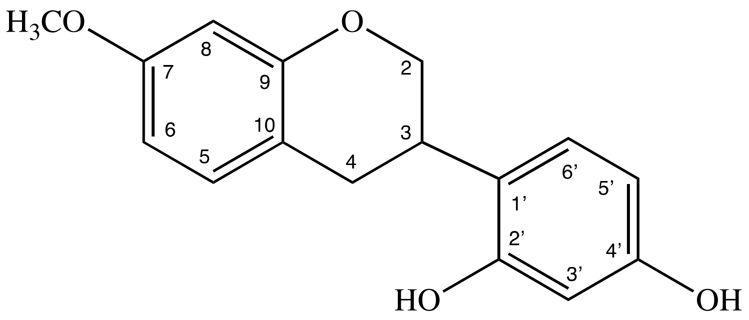

Chemical Formula: $C_{16}H_{16}O_4$

Figure S7 Structure of 2′,4′-dihydroxy-7-methoxyisoflavan (neovestitol)

Table S4: ^{1}H (400MHz), ^{13}C (100MHz) data for neovestitol in Acetone-d_6. *CD_3OD

Position	^{1}H δ ppm (mult, *J* Hz)	^{13}C δ ppm (mult)		^{1}H δ ppm* From literature [2]			^{13}C δ ppm* From literature[2]
2	4.26 (1H, ddd,10.3, 3.5, 2.0) 4.00 (1H, t, 10.1)	69.6 (CH_2)		4.32, 4.04			69.91
3	3.50 (1H, dt, 10.4, 4.3)	31.8 (CH)		3.50			31.75
4	2.98 (1H, dd, 15.6, 11.0) 2.82 (1H, ddd, 15.6, 5.3, 1.9)	30.2 (CH_2)		2.98, 2.91			30.35
5	7.07 (1H, d, 8.5)	127.9 (CH)		6.98			130.18
6	6.38 (1H, dd, 8.2, 2.5)	107.9 (CH)		6.48			107.29
7	-	159.5 (C)		-			159.14
8	6.52 (1H, d, 2.6)	101.6 (CH)		6.42			101.46
9	-	156.6 (C)		-			155.2
10	-	113.4 (C)		-			114.41
1′	-	120.1 (C)		-			120.08
2′	-	155.2 (C)		-			154.36
3′	6.31 (1H, d, 2.4)	102.8 (CH)		6.31			103.13
4′	-	155.8 (C)		-			155.11

5′	6.44 (1H, dd, 8.5, 2.6)	104.8 (CH)		6.38			108.2
6′	6.91 (1H, d, 8.2)	130.1 (CH)		6.95			128.44
7- OCH_3	3.74 (3H, s)	54.5 (CH_3)		3.77			55.35

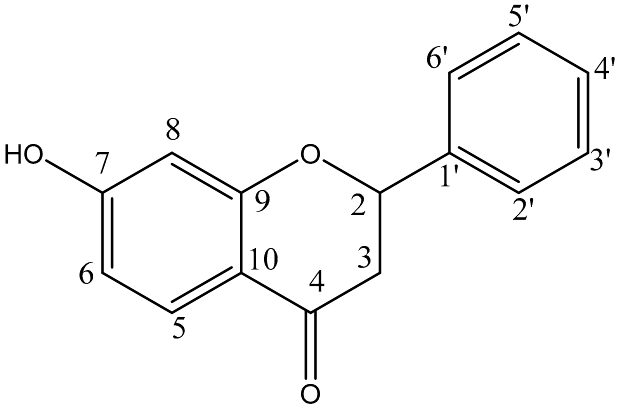

Chemical Formula: $C_{15}H_{12}O_3$

Figure S8: Structure of 7-hydroxyflavanone

Table S5: ^{1}H (400MHz), ^{13}C (100 MHz) chemical shifts for 7-Hydroxyflavanone in $CDCl_3$. *CD_3OD

Position	[1]H δ ppm (mult, *J* Hz)	[13]C δ ppm (mult)	[1]H δ ppm* From literature[3]	[13]C δ ppm* From literature[3]
2	5.46 (1H, dd, 13.2, 2.8)	80.0 (CH)	5.43	81.1
3	3.05 (1H, dd, 17.0, 13.3) 2.84 (1H, dd, 16.9, 2.9)	44.4 (CH_2)	2.96, 2.69	45.2
4	-	191.5 (C)	-	193.1
5	7.85 (1H, d, 8.7)	129.5 (CH)	7.67	129.9
6	6.55 (1H, dd, 7.5, 2.3)	111.0 (CH)	6.44	111.9
7	-	163.7 (C)	-	166.9
8	6.42 (1H, d, 2.4)	103.6 (CH)	6.32	103.9
9	-	163.9 (C)	-	165.9
10	-	114.9 (C)	-	115.1
1′	-	138.8 (C)	-	138.8
2′	7.44 (1H, m)	126.3 (CH)	7.43	127.4
3′	7.44 (1H, m)	129.0 (CH)	7.33	129.7

4′	7.38 (1H, m)	129.5 (CH)	7.33	129.6
5′	7.44 (1H, m)	129.0 (CH)	7.33	129.7
6′	7.44 (1H, m)	126.3 (CH)	7.33	127.4

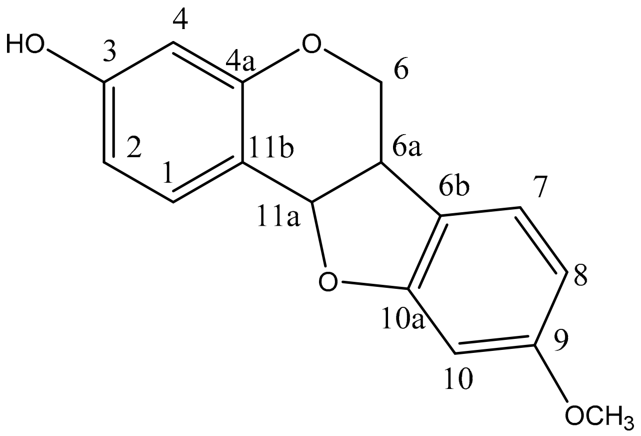

Chemical Formula: $C_{16}H_{14}O_4$

Figure S19 : Structure of medicarpin

Table S6: ^{1}H (400MHz), ^{13}C (100MHz) chemical shifts for medicarpin in $CDCl_3$. *CD_3OD

Position	Proton δ ppm (mult, *J* Hz)	Carbon δ ppm (mult)	Proton δ ppm* From literature [1,4]	Carbon δ ppm* From literature[1,4]
1	7.41 (1H, d, 8.4)	132.3 (CH)	7.31	133.2
2	6.57 (1H, dd, 8.4, 2.5)	109.5 (CH)	6.52	110.7
3	-	156.8 (C)	-	160.1
4	6.48 (1H, m)	96.9(CH)	6.33	104.1
4a	-	157.3 (C)	-	158.0
6	4.26 (1H, ddd *J* =11.04, 5.1, 0.7) 3.65 (1H, dd, 11.0)	66.7 (CH_2)	4.23, 3.59	67.6
6a	3.56 (1H, ddd, 11.1, 6.7, 5.0)	39.6 (CH)	3.57	40.9

6b	-	119.3 (C)	-	120.9
7	7.15 (1H, d , 8.8)	124.9 (CH)	7.18	126.0
8	6.49 (1H, m)	106.1 (CH)	6.4	107.3
9	-	160.8 (C)	-	162.6
10	6.44 (1H, m)	103.7 (CH)	6.46	97.6
10 a	-	161.2 (C)	-	162.0
11a	5.52 (1H, d, 6.8)	78.7 (CH)	5.48	67.6
11 b	-	112.7 (C)	-	112.9
9′- OCH_3	3.79 (3H, s)	55.7 (CH_3)	3.76	55.9

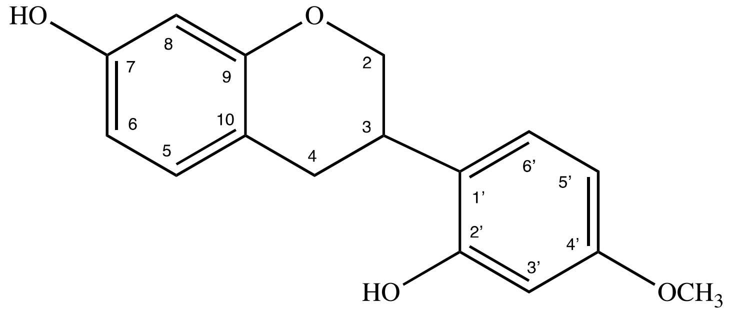

Chemical Formula: $C_{16}H_{16}O_4$

Figure S10: Structure of vestitol

Table S7 ^{1}H (400MHz), ^{13}C (100MHz) chemical shifts for vestitol in $CDCl_3$. *CD_3OD

Position	Proton δ ppm (mult, *J* Hz)	Carbon δ ppm (mult)	^{1}H δ ppm* From literature [1]	^{13}C δ ppm* From literature[1]
2	4.33 (1H, ddd, 10.4, 3.5, 2.0) 4.03 (1H, t, 10.1)	70.1 (CH_2)	4.17, 3.83	70.9
3	3.50 (1H, m)	31.9 (CH)	3.4	32.7

4	2.98 (1H, dd, 15.8, 10.4)<br>2.89 (1H, dd, 15.8, 5.4)	30.5 (CH_2)	2.81, 2.66	31.0
5	6.93 (1H, dd, 7.97, 1.08)	130.6 (CH)	6.79	131.6
6	6.39 (1H, dd, 8.5, 2.6)	108.2 (CH)	6.32	109.2
7	-	155.0 (C)	-	156.7
8	6.37 (1H, d, 2.5)	102.3 (CH)	6.25	102.3
9	-	155.2 (C)	-	156.0
10	-	114.8 (C)	-	115.4
1′	-	120.1 (C)	-	121.5
2′	-	154.5 (C)	-	156.7
3′	6.36 (1H, d, 2.6)	103.4 (CH)	6.38	101.7
4′	-	159.5 (C)	-	160.4
5′	6.47 (1H, dd, 8.5, 2.5)	106.1 (CH)	6.28	105.7
6′	7.00 (1H,d, 8.5)	128.3 (CH)	6.79	128.7
4′- OCH_3	3.76 (3H, s)	55.5 (CH_3)	3.62	55.1

1. Piccinelli, A. L.; Campo Fernandez, M.; Cuesta-Rubio, O.; Márquez Hernández, I.; De Simone, F.; Rastrelli, L. Isoflavonoids isolated from Cuban propolis. *J. Agric. Food Chem.* **2005**, *53*, 9010–9016.
2. Franchin, M.; Colón, D.F.; da Cunha, M.G.; Castanheira, F.V.; Saraiva, A.L.; Bueno-Silva, B.; Alencar, S.M.; Cunha, T.M.; Rosalen, P. Neovestitol, an isoflavonoid isolated from Brazilian red propolis, reduces acute and chronic inflammation: involvement of nitric oxide and IL-6. *Scientific Reports*, **2016**, *6*,1-12.
3. Kostrzewa-Susłow, E.; Janeczko, T. Microbial transformations of 7-hydroxyflavanone. *The Sci. World J.* **2012**, *2012*, 254929.
4. Yang, X.; Zhao, Y.; Hsieh, M.T.; Xin, G.; Wu, R.T.; Hsu, P.L.; Horng, L.Y.; Sung, H.C.; Cheng, C.H.; Lee, K.H. Total synthesis of (+)-medicarpin. *Journal of Natural products*, **2017**, *80*, 3284-3288.
